# supFunSim: Spatial Filtering Toolbox for EEG

**DOI:** 10.1007/s12021-020-09464-w

**Published:** 2020-06-21

**Authors:** Krzysztof Rykaczewski, Jan Nikadon, Włodzisław Duch, Tomasz Piotrowski

**Affiliations:** 1 Centre for Modern Interdisciplinary Technologies, Nicolaus Copernicus University, Wileńska 4, Toruń, 87-100 Poland; 2 Faculty of Mathematics and Computer Science, Nicolaus Copernicus University, Chopina 12/18, Toruń, 87-100 Poland; 3 Faculty of Humanities, Nicolaus Copernicus University, Fosa Staromiejska 1a, Toruń, 87-100 Poland; 4 Faculty of Physics, Astronomy and Informatics, Nicolaus Copernicus University, Grudziędzka 5/7, Toruń, 87-100 Poland

**Keywords:** Matlab, Toolbox, Reconstruction, Localization, Object-oriented

## Abstract

Brain activity pattern recognition from EEG or MEG signal analysis is one of the most important method in cognitive neuroscience. The supFunSim library is a new Matlab toolbox which generates accurate EEG forward model and implements a collection of spatial filters for EEG source reconstruction, including the linearly constrained minimum-variance (LCMV), eigenspace LCMV, nulling (NL), and minimum-variance pseudo-unbiased reduced-rank (MV-PURE) filters in various versions. It also enables source-level directed connectivity analysis using partial directed coherence (PDC) measure. The supFunSim library is based on the well-known FieldTrip toolbox for EEG and MEG analysis and is written using object-oriented programming paradigm. The resulting modularity of the toolbox enables its simple extensibility. This paper gives a complete overview of the toolbox from both developer and end-user perspectives, including description of the installation process and use cases.

## Introduction

Neuroimaging and signal processing methods are rapidly evolving, with the ultimate goal of reaching high time and space resolution, allowing for models of functional connectivity, activation of large-scale networks and their rapid dynamic transitions in multiple time scales. Network neuroscience is at present the most promising approach to understand the structure and functions of complex brain networks (Bassett and Sporns 2017). Electroencephalography (EEG) has excellent temporal resolution, is noninvasive and relatively easy to use. Unfortunately, signals observed at the scalp level are difficult to interpret, because they are a mixture propagated from many cortical and subcortical sources through multiple layers of the brain with several different volume conduction properties, including the scalp, skull, cerebrospinal fluid (CSF), and brain tissues. Sensors receive corrupted mixed signals from various active brain structures. Therefore, direct scalp-level EEG analysis cannot reflect the underlying neurodynamics.

Reconstruction of sources of brain’s electrical activity from EEG or magnetoencephalographic (MEG) recordings, based on spatial filters, also called “beamformers” in array signal processing, may provide meaningful information. Many papers have been written on applications of spatial filters for reliable discrimination of EEG patterns. The brain-computer interface (BCI) technology requires classification of the single-trial brain signals. Ramoser, Muller-Gerking and Pfurtscheller (Ramoser et al. 2000) used spatial filtering of a single trial EEG during imagined hand movement, achieving high classification results in discrimination of specific activation of cortical areas during left/right hand movement imagination. Spatial filters synthesized using denoising source separation can greatly reduce signal to noise (S/N) ratio (de Cheveigné and Simon 2008). Spatial filters may also be used in a single-trial neural response to maximize the S/N ratio based on a generalized eigenvalue decomposition (Das et al. 2020). Optimal spatial filters were designed to detect high-frequency visual evoked potentials for BCI applications (Molina and Mihajlovic 2010). Adaptive common spatial pattern patches provide highly distinctive features without large amount of training data (Sannelli et al. 2016).

Long continuous EEG recordings are contaminated by many physiological artifacts, such as eye blinks, muscular movements, cardiac activity or electrode movements. Spatial filters are also used to clean artifacts recovering relevant brain activity in clinical applications, localizing sources of epileptic disorders for planning medical or surgical treatment. Ille et al. (2002). Beamforming may also help to account for microsaccades that distort higher frequency EEG components (Craddock et al. 2016). Clearly the importance of spatial filtering cannot be overstated.

Creation of spatial filters requires solving forward and inverse problems in signal analysis. There are several libraries implementing various forward and inverse solutions, see for example (Oostenveld et al. 2011a; Tadel et al. 2011; Gramfort et al. 2014). However, to the best of our knowledge, none of them implements up-to-date state-of-the-art spatial filters based on high precision EEG forward models. supFunSim Matlab toolbox (library) described in this paper is an open source software that fills this gap by providing, among others, implementation of various forms of the linearly constrained minimum-variance filters (LCMV) (Frost 1972; Van Veen et al. 1997; Sekihara and Nagarajan 2008), eigenspace LCMV (Sekihara and Nagarajan 2008), nulling (NL) (Hui et al. 2010), and minimum-variance pseudo-unbiased reduced-rank (MV-PURE) filters (Piotrowski and Yamada 2008; Piotrowski et al. 2009; Piotrowski et al. 2019). It also enables source-level directed connectivity analysis using partial directed coherence (PDC) (Baccala and Sameshima 2001a) and directed transfer function (Kamiński et al. 2001) measures by applying it to the time series representing reconstructed activity of sources of interest.

The supFunSim toolbox is based on FieldTrip (Oostenveld et al. 2011a), an excellent Matlab toolbox for EEG and MEG signal analysis. It can be used as an extension (plugin) to FieldTrip. The code of presented library was created to perform experiments for the paper (Piotrowski et al. 2019) and later refactored and reimplemented in an object-oriented way. The source code of the toolbox is publicly available at https://github.com/nikadon/supFunSim as an Org-mode file, Jupyter notebook, and also as a plain Matlab source code.

The library was written paying attention to its modularity and possibilities of further development. It is segmented into separate components based on their functionality. Functions are not precompiled, as script libraries have the advantage of being easily maintainable and extensible. In this way users can easily extend the existing functionality by implementing new algorithms or extending those already implemented.

The paper is organized as follows. First, we briefly introduce the forward and inverse problems in EEG (similar considerations also apply to MEG). Then, we discuss the benefits of the object-oriented approach for our toolbox and its extensibility. We close with the use cases which appear frequently in practical applications of this toolbox. In appendices we provide mathematical details of the implemented spatial filters and the full list of toolbox parameters.

## EEG Measurement Model

Electromagnetic signals that originate within *enchanted loom*^1^ of human brain volume are propagated through head compartments (Mosher et al. 1999). This *dissolving pattern* of brain electrical activity can be detected on the surface of scalp using electroencephalography (EEG).

At a given time instant the EEG data acquisition can be well approximated by a linear equation of the generic form

1 $$  y = H(\theta)q + H_{\mathfrak{i}}(\theta_{\mathfrak{i}})q_{\mathfrak{i}} + H_{\mathfrak{b}}(\theta_{\mathfrak{b}}) q_{\mathfrak{b}} + n_{\mathfrak{m}}, $$

where, for *m* EEG sensors located at the scalp and *l* sources of interest modelled as equivalent current dipoles (ECD) located inside brain’s volume,

- by $y \in \mathbb {R}^{m}$ we denote the signal observed in the sensor space at a given time instant,
- $\theta = (\theta _{1}, \ldots , \theta _{l}) \in {\Theta } \subset \mathbb {R}^{l \times 3}$ represents locations of the sources of interest, i.e., for the *i*-th source the vector representing its location is $\theta _{i} \in \mathbb {R}^{3}.$ Here, Θ denotes the set of all subsets of locations of source signals,
- $H(\theta ) \in \mathbb {R}^{m \times l}$ is the sensor array response (*lead-field*) matrix of the sources of interest,
- $q\in \mathbb {R}^{l}$ is a vector of electric activity of the *l* sources of interest representing magnitudes of ECDs,
- similarly, for the *k* interfering noise sources, $\theta _{\mathfrak {i}} = \left ({\theta }_{1}^{\mathfrak {(i)}}, \ldots , {\theta }_{k}^{\mathfrak {(i)}}\right ) \in \mathbb {R}^{k \times 3}$ are the interference source locations, $H_{\mathfrak {i}}(\theta _{\mathfrak {i}}) \in \mathbb {R}^{m \times k}$ is the corresponding lead-field matrix, $q_{\mathfrak {i}} \in \mathbb {R}^{k}$ is the corresponding interference activity,
- for the *p* sources of background activity of the brain $\theta _{\mathfrak {b}} := \left (\theta _{1}^{\mathfrak {(b)}}, \ldots , \theta _{p}^{\mathfrak {(b)}}\right ) \in \mathbb {R}^{p \times 3}$ are the background source locations (i.e. sources which are not sources of interest and are uncorrelated with them), $H_{\mathfrak {b}}(\theta _{\mathfrak {b}}) \in \mathbb {R}^{m \times p }$ is the corresponding lead-field matrix, $q_{\mathfrak {b}} \in \mathbb {R}^{p}$ is the corresponding background activity,
- $n_{\mathfrak {m}} \in \mathbb {R}^{m}$ is an additive white Gaussian noise (AWGN) interpreted as a measurement noise present in the sensor space.

Equation 1 represents a single sample of EEG data *y* from subjects’ scalp at a given time (*forward solution*). It enables, through customizable parameters, accurate modelling of real-world EEG experiments. We shall emphasize at this point that not all components of the model in Eq. 1 have to be considered, i.e., one may select only those signal components that fit the aims of user’s simulation settings.

The lead-field matrices establishing signal propagation model are estimated on the basis of geometry and electrical conductivity of head compartments together with position of sensors on the scalp. We consider these properties to be fixed in time during a single EEG data acquisition session. Therefore, lead-field-matrices are assumed to be time-invariant in such circumstances (its values do not change during acquisition time in a single session). We also note that the above EEG forward model (1) assumes that the orientations of the ECD moments are fixed during measurement period, and only their magnitudes $q,q_{\mathfrak {i}},q_{\mathfrak {b}}$ vary in time. We also assume that orientations of the ECD moments are normal and directed outside with respect to the cortical surface mesh. This is in accordance with the widely recognized physiological model of EEG signal origin that considers pyramidal cortical neurons to be the main contributor to the brain’s bioelectrical activity that can be measured on the human scalp (Baillet et al. 2001).

We assume that *q* and $q_{\mathfrak {i}}$ may be correlated (i.e., that source of interest can interfere with each other), but are uncorrelated with the background sources $q_{\mathfrak {b}}$ and the noise $n_{\mathfrak {m}}.$ We further assume that *q*, $q_{\mathfrak {i}}$, $q_{\mathfrak {b}}$, $n_{\mathfrak {m}}$ are zero-mean weakly stationary stochastic processes with the exception that *q* may contain in addition a deterministic component simulating evoked (phase-locked) activity in event-related EEG experiments. In our toolbox the presence of this component is controlled by the SETUP.ERPs variable.

## EEG Source Reconstruction

**Fig. 1:**
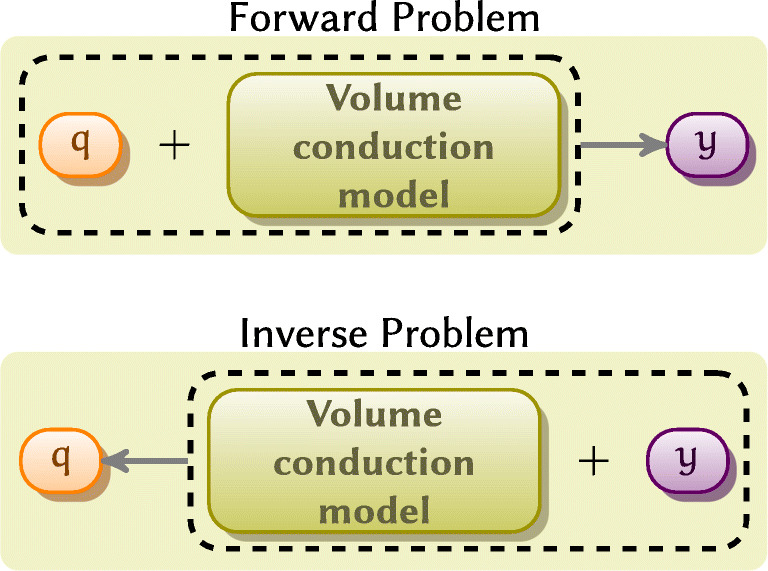
The relationship between forward and inverse problems

Having solved the EEG forward problem which introduced, in particular, the lead-field matrices embodying the propagation model of brain’s electromagnetic activity, we are in a position to solve the inverse problem. Here it amounts to reconstruction of time courses of activity of sources at predefined locations. That means that we assume that the locations of the sources of interest *θ* are known. This can be achieved by defining regions of interest using source localization methods, e.g., minimum-norm (Pascual-Marqui 1999) or spatial filtering-based methods (Moiseev et al. 2011; Piotrowski and Nikadon 2020), or referring to neuroscience studies that have identified regions of interest as in paper by Hui et al. (Hui et al. 2010). Then, the goal is to reconstruct the activity *q* of sources of interest based on the observed signal *y* as

2 $$ \widehat{q} = Wy, $$

where $W \in \mathbb {R}^{l \times m}$ is a matrix representing spatial filter’s coefficients. The definitions of the filters currently implemented in the toolbox are given in A*Implemented spatial filters* (Fig. 1).

## Toolbox Signal Processing Outline

### Overview

In order to obtain EEG signal *y* we need first to generate source activity signals and propagate it to electrodes according to the forward model given in Eq. 1. The source signals are generated using the method described in Franaszczuk et al. (1985), which uses stable multivariate autoregressive (MVAR) model with predefined coefficient matrices. This results in a wide-sense stationary signals generated with predefined pairwise linear dependencies (correlations). Such approach has been studied and used in literature, see, e.g., Haufe (2012), Baccalá and Sameshima (2001b), and Neumaier and Schneider (2001), and is specially useful in investigating functional dependencies between activity of sources using directed connectivity measures such as partial directed coherence (PDC) (Baccala and Sameshima 2001a) or directed transfer function (DTF) (Kuś et al. 2004).

Gaussian pseudo-random vectors simulating autoregressive processes are generated using the arsim function available from Schneider and Neumaier (2001). In this way, we obtain multivariate times series representing activity of sources of interest *q* (denoted in the code as sim_sig_SrcActiv.sigSRC), sources of interference noise $q_{\mathfrak {i}}$ (sim_sig_IntNoise.sigSRC), and sources of background activity $q_{\mathfrak {b}}$ (sim_sig_BcgNoise.sigSRC).

Moreover, as our framework allows to add event-related potentials (ERP, flag variable SETUP.ERPs in the code) to the source signal, each source activity is divided into *pre* and *pst* parts in relation to the onset of event (*q* into *q*_*pre*_ and *q*_*pst*_, etc.) in order to enable simulation of ERP experiments. In particular, the ERP signal may be added to *q*_*pst*_, but not to *q*_*pre*_.

Furthermore, the *pre* and *pst* subsignals are used to implement spatial filters. In particular, noise correlation matrix *N* may be estimated from *y*_*pre*_ signal and signal correlation matrix *R* may be estimated from *y*_*pst*_ signal.

The *y*_*pst*_ signal is also used for evaluation of the fidelity of reconstruction, as a filter *W*_*f*_ produces an estimate of the activity signal source $\widehat {q}_{pst, f}$ based on *y*_*pst*_. Then, an MVAR model is fitted to the reconstructed source activity using arfit function, yielding reconstructed composite MVAR model matrix *A*00_*f*_. This matrix can then be used to investigate directed connectivity using partial directed coherence (PDC) (Baccala and Sameshima 2001a) and directed transfer function (DTF) (Kamiński et al. 2001).

**Fig. 2:**
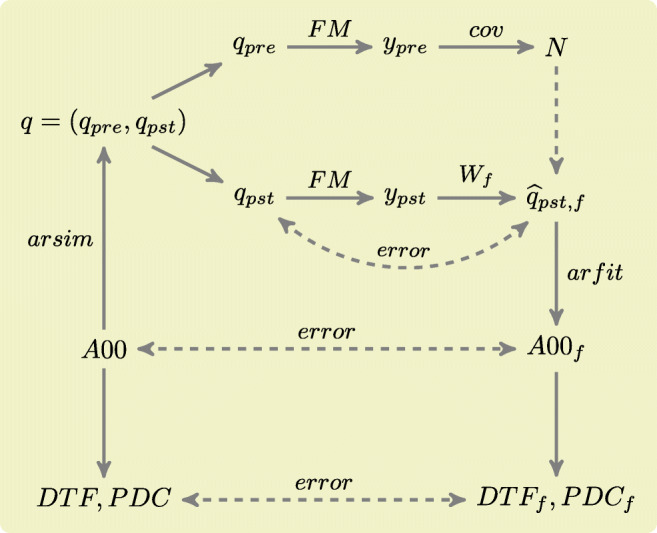
Overview of processing of signals by the toolbox

The overview of signals processed by the toolbox and dependencies between them is depicted in Fig. 2.

### Brain Signals in Source Space

Items 1 and 2 below detail generation of source signals $q,q_{\mathfrak {i}}$ and $q_{\mathfrak {b}}.$ Item 3 concerns definition of volume conduction model. The computation of lead-field matrices *H*, $H_{\mathfrak {i}}$ and $H_{\mathfrak {b}}$ is discussed in the subsequent Section *Brain Signals in Sensor Space*.

1. **Positioning sources:**
  File supFunSim/mat/sel_atl.mat contains 15000 vertex FreeSurfer (Dale et al. 1999) brain tessellation together with atlases (Dale et al. 1999; Fischl et al. 2004) that provide parcellation of the mesh elements into cortical patches (regions of interest, ROIs). This file was provided with the BrainStorm toolbox (Tadel et al. 2011). First, we randomly select an arbitrary number of ROIs by selecting items from Destrieux and Desikan-Killiany atlases (Fischl et al. 2004; Desikan et al. 2006). In each ROI, each vertex is a candidate node for location of the dipole source. Then, an arbitrary number of locations can be drawn within each ROI separate for *q*, $q_{\mathfrak {i}}$ and $q_{\mathfrak {b}}$.
  Most of the simulation parameters are controlled using SETUP structure. The geometrical arrangement and number of cortical sources in each ROIs is controlled using SRCS field (SETUP.SRCS), which is a three-column <int> array, where:
  rows represent consequent ROIs (thus, the number of rows determines the number of ROIs used in the simulations),
  the first column represents sources of interest, the second column represents sources of interference, and the third column represents sources of background activity,
  integer values of this array represent number of sources in the given ROI for the given signal type.
  For the end-user this provides mechanism not only to control the total number of sources of a particular type, but also to choose their spatial distribution. Additionally, we provide a mesh representing both *thalami* (jointly) as a structure containing potential candidates for the non-cortical (deep) sources of signal/noise. Variable SETUP contains also the field (SETUP.DEEP) which defines the number of signals in the brain center (around thalami) belonging to a particular signal type (of interest, interfering, or background activity). Furthermore, in order to account for the mislocalization of sources, together with the original lead-fields we also generate *perturbed* lead-fields for the source activity reconstruction. These are generated using locations that are shifted with relation to the original locations and direction that is rotated in relation to the original (normal to cortex surface) dipole orientation. Default shift is random and < 5 mm in each direction (*x*, *y*, *z*). Default rotation is random, bounded by $\frac {\pi }{32}$ (azimuth and elevation).
2. **Sources Timecourse:**
  Following (Neumaier and Schneider 2001; Schneider and Neumaier 2001), we use stable MVAR model to generate time-series. It is assumed that such model generates a realistic source activity (Korzeniewska et al. 2003). We create separate models for time-series *q* and $q_{\mathfrak {b}}$. The $q_{\mathfrak {i}}$ is obtained as a negative of *q* with Gaussian uncorrelated noise added with the same power as the *q*, i.e. $q_{\mathfrak {i}} = -q + n_{\mathfrak {i}}$. In this way, we obtain correlated time-series *q* and $q_{\mathfrak {i}}$.
  The *l*-variate autoregressive model of order *p* for a stationary time-series of state vectors ${{q}^{n}} \in \mathbb {R}^{l}$ is defined at time instant *n* as
  3 $$ {{q}^{(n)}} = \sum\limits_{s = 1}^{p}  {{A}_{s}}{{q}^{(n - s)}} + {{\varepsilon}_{n}},  $$
  where *q*^(*n*)^ is the state vector at time *n* *p* is the order of the model of order *p* = 6 by default, matrices ${{A}_{1}}, \ldots , {{A}_{p}} \in \mathbb {R}^{l \times l}$ are the coefficient matrices of the AR model, and *ε*_*n*_ is the *l*-dimensional additive white Gaussian noise (Haufe 2012). For the signal of interest *q*, we also give the possibility to include deterministic component simulating evoked (phase-locked) activity in event-related EEG experiments. The presence of this component is controlled by the SETUP.ERPs variable. Then, *q* = *q*^(*n*)^ + *q*^(*d*)^, where *q*^(*d*)^ is the deterministic ERP component. In the toolbox, *q*^(*d*)^ is generated using Matlab gauswavf function generating 1st derivative of Gaussian wavelet function (Řondík et al. 2011).
  Similarly, $q_{\mathfrak {b}}$ is simulated using independent, random and stable MVAR model (of order *r* = 6 by default):
  4 $$ {{q}^{(n)}_{\mathfrak{b}}} = \sum\limits_{s = 1}^{r}  {{B}_{s}}{{q}^{(n - s)}_{\mathfrak{b}}} + {{\varepsilon}_{n}^{\mathfrak{b}}}. $$
  MVAR model is considered to be *stable* if the absolute values of all eigenvalues of all matrices *A*_*s*_ (respectively, *B*_*s*_) are less than one. We used procedure adapted from Gómez-Herrero et al. (2008) (namely, we adapted the stablemvar function) to generate stable MVAR model that was used for times-series generation. During generation of MVAR models for *q* and $q_{\mathfrak {b}}$, the coefficient matrix *A*_*s*_ (respectively, *B*_*s*_) is multiplied by a masking matrix that has 80 % (by default) of its off-diagonal elements equal to zero. All the remaining diagonal and off-diagonal masking coefficients are equal to one. In code, the composite MVAR model matrix is represented by the variable A00, see Fig. 3. Such procedure allows, in particular, to obtain specific profile of directed dependencies between activity of sources of interest. This approach is taken from Baccalá and Sameshima (2001b). Moreover, it gives us the possibility to implement the directional measures of casual dependencies: PDC and DTF measures. Namely, partial directed coherence and directed transfer function are matrices defined using Fourier transform of MVAR model (3), i.e.
5where is the identity matrix, *λ* is normalized frequency, |*λ*|≤ 0.5. Then, partial directed coherence between *i*-th and *j*-th signals is given by Baccala and Sameshima (2001a)
  6 $$ \text{PDC}_{ij}(\lambda) := \frac{ A_{ij}(\lambda) }{\sqrt{a^{*}_{j}(\lambda) a_{j}(\lambda)}} = \frac{ A_{ij}(\lambda) }{\sqrt{ {\sum}_{i=1}^{l}\left| A_{ij}(\lambda) \right|^{2} }}, $$
  where *A*_*ij*_(*λ*) is *ij* element of matrix *A*(*λ*), *a*_*j*_(*λ*) is *j* th column of *A*(*λ*) and ∗ means Hermitian transpose. It takes values in the interval [0,1] and measures the relative strength of the interaction of a given source signal *j* to source signal *i* normalized by strength of all of *j*’s connections to other signals (Blinowska and Zygierewicz 2011).

**Fig. 3:**
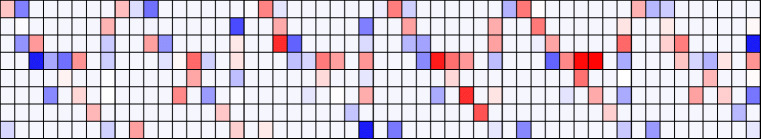
A00: the original coefficient matrix used for time-series generation, with sample values *l* = 9 and *p* = 6., after application of a random mask
  Directed Transfer Function (DTF) is defined as
  7 $$ \text{DTF}_{ji}(\lambda) := \frac{ H_{ji}(\lambda) }{\sqrt{{\sum}_{i=1}^{l}\left| H_{ji}(\lambda) \right|^{2}}}, $$
  where *H*(*λ*) := (*I* − *A*(*λ*))^− 1^ is the **transfer matrix**, *i*, *j* = 1, … , *l*. It can be interpreted as ratio between inflow from channel *i* to chanel *j* nomalized by sum of inflows to channel *j*. As an alternative to the generation of simulated data we also provide an example script that demonstrates how to use real data in EEGLAB format to test the performance of spatial filters.
3. **Volume conduction model:**
  We used FieldTrip (FT) toolbox (Oostenveld et al. 2011b) to generate volume conduction model (VCM) and lead-field matrices. VCM was prepared using ft_prepare_headmodel function implementing DIPOLI method (Oostendorp and Van Oosterom 1989) which takes as arguments three triangulated surface meshes representing the outer surfaces of brain, skull and scalp supFunSim/mat/msh.mat (Tadel et al. 2011). VCM is available with our simulation precomputed (supFunSim/mat/sel_vol.mat), although if required, FieldTrip toolbox allows for easy computation of custom VCMs on the basis of triangulated meshes which can be obtained from structural (T1) MRI scans.
  The default head geometry is based on the Colin27 2 (Tadel et al. 2011; Holmes et al. 1998; Aubert-Broche et al. 2006). However, it can be easily substituted at user’s discretion by replacing triangulation meshes stored in SETUP.sel_msh (a list of structures containing: scalp outer mesh, skull outer mesh and skull inner mesh, where the last triangulation represents “rough” brain outer mesh). Common choices include realistic head models generated on the basis of structural MRI scans or spherical models.

### Brain Signals in Sensor Space

In sensor space, we need to provide positions for the electrodes (*a.k.a.* sensor montage). By default, in our simulations we use *HydroCel Geodesic Sensor Net* utilizing 128 channels as EEG cap layout. Other caps can easily be used by substituting content of the supFunSim/mat/sel_ele.mat with electrode position coordinates obtained either from specific EEG cap producer or from standard montages that are available with EEG analysis software such as EEGLAB (Delorme and Makeig 2004) or FieldTrip (Oostenveld et al. 2011b). Additionally, for real data acquisition setup, the electrode positions can be captured using specialized tracking system for every EEG session.

The volume conduction model, together with source locations and their orientations, are obtained as described in the three points of the previous section. Together with electrode positions, they are the input arguments for the ft_prepare_leadfield function which during simulations is run three times outputting *H*, $H_{\mathfrak {i}}$ and $H_{\mathfrak {b}}$.

## Implementation Details

### Object-Oriented Approach

The object-oriented approach provides the toolbox with several desirable properties of the code and avoids drawbacks of standard procedural approach commonly employed in Matlab scripts. For example, Matlab by default stores all variables in one common workspace. This causes bugs in the code that may be hard to detect. On the other hand, the object-oriented approach circumvents this difficulty by its inherent encapsulation property, enclosing variables within a class and sealing it securely from the outside environment. We also note that the construction of the Matlab language requires explicit assignment of an instance of the class each time a method acts on it. Such approach necessitates language constructs such as obj = obj.method, where obj is a given instance of a class.

Data structures created during simulation can be accessed interactively in the Jupyter notebook or in Matlab script. In particular, property MODEL from EEGReconstruction class contains information about all variables used within the simulation pipeline.

### Benefits of Literate Programming

**Fig. 4:**
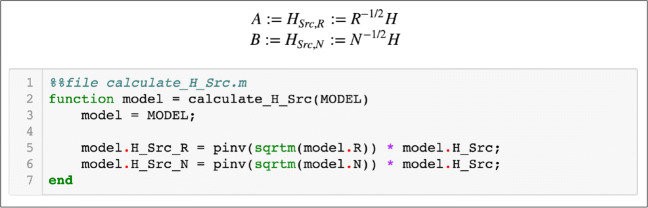
Example of Jupyter notebook

The code of the toolbox was written in Jupyter, which is an open source application that allows users to create interactive and shareable notebooks. Jupyter allows for easy export of whole documents to HTML, LATE X, PDF and other formats and is a very convenient tool for academic prototyping, because it permits comments in the code using LATE Xmathematical expressions. The source code blocks are interspersed with ordinary natural language blocks that provide explanations and some insights explaining the intrinsic mechanics of the code. Such an approach is called *literate programming* (Knuth 1984). An example of mathematical comment and corresponding code is given in Fig. 4.

However, using Jupyter is not necessary to run the toolbox. Instead, the code can be executed under powerful and cross-platform Matlab environment. To that end we have prepared a version of the toolbox as the set of Matlab files stored in supFunSim.zip archive.

The toolbox does not have a GUI (Graphical User Interface). Instead, user interacts with it using provided functions. Therefore, as a prerequisite to use the supFunSim toolbox knowledge of Matlab language basics is required.

### Installation

Installation of the supFunSim is independent of the operating system. For a simple installation similar to FieldTrip’s installation process, the user can download the file supFunSim.zip from https://github.com/nikadon/supFunSim. This archive contains the whole toolbox. After unpacking this archive, the user should execute addpath(genpath('/path/to/toolboxes/ supFunSim/'])). Function genpath will ensure that all subdirectories will be added to your path. It is most convenient to have the addpath function in the startup.m script located in the Matlab directory. Then, the user may run the RunAll.m script (preferably line by line, in order to follow execution). The user has to make sure that there is a mat/ directory (or a link to it) containing mat files required by the toolbox in the toolbox directory. The mat files are available for download at http://fizyka.umk.pl/~tpiotrowski/supFunSim. s More advanced user may manipulate Jupyter notebooks directly and use make tool to set up the toolbox from scratch. Namely, in order to open and run notebooks the user should download and install Jupyter notebook with Matlab kernel. The easiest way to do it (under a Unix-like system) is by executing the following instructions in the command line. First, we set up a virtual environment, which will install Python packages locally: 
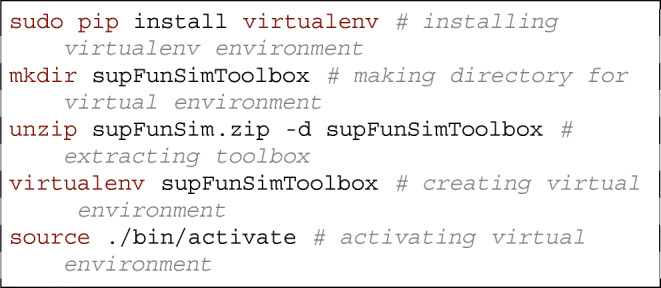


Next, we install all necessary packages and install Matlab Engine API for Python 
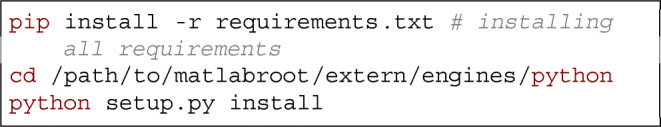


We also provide a make tool for simple administration of notebooks’ code. For example, the user may execute makeeverything in terminal in order to generate all source code files. See README.md file in the repository for details.

At this stage, one can run the simulations and “play with” the code by going to supFunSimToolbox directory and running 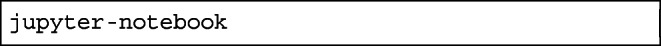


Finally, the description of the installation under Windows can be found in the README.md file.

### Prerequisites/Dependencies

Beyond Matlab our toolbox requires the following dependencies: FieldTrip toolbox (version 20150227) (Oostenveld et al. 2011a), MVARICA toolbox (version 20080323) (Gómez-Herrero et al. 2008), ARfit toolbox (version 20060713) (Neumaier and Schneider 2001; Schneider and Neumaier 2001). Location of these toolboxes should be added to Matlabpath.

## Application Structure

The simulation framework provided with the current paper consists of a set of modules represented by corresponding classes. The classes are defined in separate (self-contained) notebooks. The classes depend on auxiliary functions generated alongside with them when appropriate make target is invoked. In this way, a given class is enclosed and all operations involving it are made within it. The toolbox contains six classes (described in the next section) with a number of auxiliary functions.

### Overview of Toolbox Classes

The main functionality of the toolbox is provided by the following five classes:

- EEGParameters.ipynb — class generating parameters for simulations. It can be overwritten in order to obtain desired parameters for a sequence of simulations.
- EEGSignalGenerator.ipynb — class used to generate signal for forward modelling of sources. It can be overwritten to generate a signal with given or desired properties.
- EEGForwardModel.ipynb — class implementing forward model. In constructs and adds together all signals (source activity, background activity and interference noise). Furthermore, the lead-field matrix is build using FieldTrip library based on the selected head model.
- EEGReconstruction.ipynb — class implementing methods used in the reconstruction of the underlying neuronal activity. All spatial filters are implemented in this class.
- EEGPlotting.ipynb — class implementing plots detailing execution of experiments. Various visualizations are accessible through the methods included in this class.

We also wrote a class for unit testing of the toolbox functionality:

- EEGTest.ipynb — class implementing unit tests and validation of the code against the functional-code toolbox implementation.

**Fig. 5:**
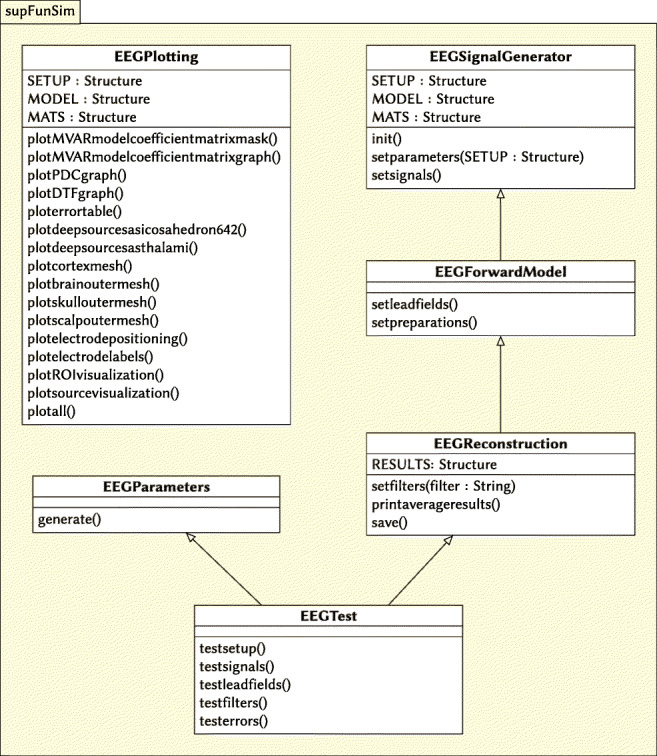
Dependencies between classes

Figure 5 gives an overview of relationships between implemented classes. We should also emphasize that modular design facilitates reuse and extensibility of the source code and adaptation to other applications. For example, if one wishes to generate source signals in a different way compared with the implemented version, one needs only to overwrite EEGSignalGenerator class (or some of its methods) while keeping the rest of the code and its functionality intact.

### Mat Files

Directory mat/ contains third-party data with the geometry of the brain, taken from the Brainstorm toolbox (Tadel et al. 2011) and extracted using FieldTrip procedures:

- sel_msh.mat — head compartments geometry (vertices and triangulation forming meshes for brain, skull and scalp); this data can be used as an input for volume conduction model and lead-field generation using FieldTrip (or any other toolbox that can be used to generate forward model);
- sel_vol.mat — volume conduction model (head-model). This structure contains head compartments geometry (the same as in sel_msh.mat) accompanied by their conductivity values and a matrix containing numerical solution (utilizing boundary or finite element method) to a system of differential equations describing propagation of the electric field. This data is obtained using FieldTrip’s dipoli method and is used as an input to the function that calculates the lead-field matrix. The default volume conduction model was prepared in accordance with the instruction provided in the FieldTrip tutorial *Creating a BEM volume conduction model of the head for source-reconstruction of EEG data* available at Knuth (2016). This structure is used to compute lead-fields;
- sel_geo_deep_thalami.mat — mesh containing candidates for location of deep sources (based on *thalami*). The mesh was prepared on the basis of the Colin27 (Tadel et al. 2011; Holmes et al. 1998; Aubert-Broche et al. 2006) MRI images;
- sel_geo_deep_icosahedron642.mat — mesh containing candidates for location of deep sources (based on *icosahedron642*);
- sel_atl.mat — cortex geometry with (anatomical) ROI parcellation (cortex atlas). This detailed triangulation is parceled into cortical patches (a.k.a. regions of interest, ROIs). It contains a 15000 vertices and it is based on the sample data accompanying the BrainStorm toolbox (Tadel et al. 2011). It was originally prepared using FreeSurfer (Dale et al. 1999; Fischl et al. 2004) software;
- sel_ele.mat — geometry of electrode positions. By default we use *HydroCel Geodesic Sensor Net* sensor montage utilizing 128 channels available. The electrode positions file is available with the FieldTrip toolbox as GSN-HydroCel-128.sfp file;
- sel_src.mat — lead-fields of all possible source locations.

#### Simulation Parameters Class

This class is responsible for setting up parameters of simulations.

- EEGParameters:
  - generate — this method generates the set of parameters of simulations. The method itself is mainly based on generatedummysetup function which itself uses setinitialvalues and setsnrvalues functions containing default configuration for the reconstruction. Users willing to change basic configuration should edit the configurationparameters.m file. The assignments of parameters’ values made in this file overwrite default parameters’ settings.
    For the complete list of all simulation parameters consult Table 1.
    For unit testing, configuration from the testparameters should be used. The configuration in this file agrees with the configuration used in supFunSim.org file. To perform unit testing, simply uncomment appropriate line in the generatedummysetup.m file.

**Table 1:** SETUP configuration

Parameter	Description
rROI	random (if 1) or predefined (if 0) ROIs
rPNT	random (if 1) or predefined (if 0) candidate points for source locations
SRCS	represent SrcActiv, IntNoise and BcgNoise, respectively
AzElSrcS,AzElIntS,AzElBcgS	represent deviation of azimuth and elevation (in radians) from the orthonormal for the SrcActiv, IntNoise and BcgNoise, respectively
DEEP	deep sources
ERPs	add ERPs (timelocked activity)
n00	number of time samples per trial
K00	number of independent realizations of signal and noise based on generated MVAR model
P00	order of the MVAR model used to generate time-courses for signal of interest
FRAC	proportion of ones to zeros in off-diagonal elements of the MVAR coefficients masking array
STAB	VAR stability limit for MVAR eigenvalues (less than 1.0 results in more stable model producing more stationary signals (Neumaier and Schneider 2001))
RNG	range for pseudo-random sampling of eigenvalues for MVAR coefficients range
ITER	iterations limit for MVAR pseudo-random sampling and stability verification
PDC_RES	frequency resolution vector for normalized PDC and DTF estimation
TELL	provide additional comments during code execution (“tell me more”)
PLOT	plot figures during the intermediate stages
SCRN	get screens positions
DISP	force figures to be displayed on (3dr) screen
SEED	seed for random number generation
SEEDS	hardcoded seeds to ensure repeatability of the simulation
RANK_EIG	rank of EIG-LCMV filter: set to number of active sources
fltREMOVE	to keep (if 0) or remove (if 1) selected filters
SHOWori	to show (if 1) or do not show (if 0) Original and Dummy signals on figures
IntLfgRANK	rank of patch-constrained reduced-rank lead-field
supSwitch	rec: run reconstruction of sources activity, loc: find active sources
thalamus	type of head model
DEBUG	if we want to debug
PATH	path to directory with the code
SRATE	sampling rate
CUBE	perturbation of the lead-fields based on the shift of source location within a cube of given edge length (centered at the original lead-fields locations)
CONE	perturbation of the lead-fields based on the rotation of source orientation (azimuth TH, elevation PHI)
H_Src_pert	use original (if 0) or perturbed (if 1) lead-field for signal reconstruction
H_Int_pert	use original (if 0) or perturbed (if 1) lead-field for nulling constrains
SINR	signal to interference noise power ratio expressed in dB (both measured on electrode level)
SBNR	signal to biological noise power ratio expressed in dB (both measured on electrode level)
SMNR	signal to measurment noise power ratio expressed in dB (both measured on electrode level)
WhtNoiseAddFlg	white noise admixture in biological noise interference noise (FLAG)
WhtNoiseAddSNR	SNR of BcgNoise and WhiNo (dB)
SigPre	final signal components for pre-interval (use zero or one for signal)
IntPre	final signal components for pre-interval (use zero or one for interference noise)
BcgPre	final signal components for pre-interval (use zero or one for background activity)
MesPre	final signal components for pre-interval (use zero or one for measurement noise)
SigPst	final signal components for post-interval (use zero or one for signal)
IntPst	final signal components for post-interval (use zero or one for interference noise)
BcgPst	final signal components for post-interval (use zero or one for background activity)
MesPst	final signal components for post-interval (use zero or one for measurement noise)
DATE	date
NAME	temporary file name
SINR_RNG	range of SNR for interference signals
SBNR_RNG	range of SNR for background signals
SMNR_RNG	range of SNR for measurment noise

#### Signal Generation Class

This class is responsible for EEG signal generation.

- EEGSignalGenerator:
  - init — this method initializes all toolboxes required by supFunSim. It sets path to toolboxes, creates their default settings, sets head model, geometry of patches etc.;
  - setparameters — this method sets configuration for simulation using parameters from EEGParameters class;
  - setsignals — this method generates all source-level signals: sim_sig_SrcActiv.sigSRC, sim_sig_IntNoise.sigSRC, sim_sig_BcgNoise.sigSRC, as well as sensor level sim_sig_MesNoise.sigSNS measurement noise:
    - makeSimSig — MVAR-based signal generation; the signal is then halved into *pre* and *pst* parts,
    - generatetimeseries sourceactivity — generates sim_sig_Src_Activ.sigSRC signals of sources of interest using makeSimSig and if required, adds ERP deterministic signal to the *pst* part of the signal of interest,
    - generatetimeseries interferencenoise — generates sim_sig_IntNoise.sigSRC interference noise signal as the negative of sim_sig_SrcActiv.sigSRC signal of interest with added white Gaussian noise of prescribed power relative to the power of sim_sig_SrcActiv.sigSRC,
    - generatetimeseries backgroundnoise — generates sim_sig_BcgNoise.sigSRC background activity signal using makeSimSig,
    - generatetimeseriesmeasure ment noise — generates sim_sig_MesNoise.sigSNS measurement (sensor-level) noise signal as an additive white Gaussian noise.

#### Forward Model Class

Class EEGForwardModel generates solution to the forward problem: leadfield matrices and the resulting electrode-level signal.

- EEGForwardModel:
  - setleadfields — this method generates leadfield matrices:
    - geometryrandomsampling — random or user-defined selection of cortex ROIs for sources (of interest, interfering activity, background activity),
    - geometryindices — identification of cortex ROIs’ indices within cortex atlas,
    - geometrycoordinates — coordinates of vertices of sources within selected ROIs,
    - geometrydeepsources — coordinates of vertices of sources within thalamus,
    - geometryperturbation — generation of perturbed source locations and orientations,
    - geometryleadfieldscomput ationy — computation of original lead-fields of sources of interest sim_lfg_SrcActiv_orig.LFG, sources of interfering activity sim_lfg_IntNoise_orig.LFG, sources of background activity sim_lfg_BcgNoise_orig.LFG, as well as their respecitve perturbed versions sim_lfg_SrcActiv_pert.LFG, sim_lfg_IntNoise_pert.LFG, sim_lfg_BcgNoise_pert.LFG, respectively,
    - forwardmodeling — multiplication of source signals by their corresponding lead-field matrices yielding sensor-level signals of sources of interest sim_sig_SrcActiv.sigSNS, sources of interfering activity sim_sig_IntNoise.sigSNS, and sources of background activity sim_sig_BcgNoise.sigSNS;
  - setpreparations — generates output EEG signal and prepares for signal reconstruction using spatial filters:
    - preparationsnrsadjustment — rescales sensor-level signals to the appropriate SNRs,
    - prepareleadfieldconfigu- ration — determines whether original or perturbed lead-field matrices of sources of interest and of interfering sources will be made available to spatial filters according tu user’s setting of SETUP.H_Src_pert and SETUP.H_Int_pert flags; moreover, reduces the rank of lead-field matrix of interfering sources according to SETUP.IntLfgRANK variable value,
    - preparemeasuredsignals — sums sensor-level signals and adds measurement noise signal (sim_sig_MesNoise.sigSNS) to produce output y_Pre and y_Pst EEG signals.
    - store2eeglab — a method that allows the user to save data in the EEGLAB format; this toolbox and its plugins allows for better preprocessing and visualization of EEG signal.
    - rawAdjTotSNRdB — adjusts power of sim_sig_IntNoise.sigSNS, sim_sig_BcgNoise.sigSNS and sim_sig_MesNoise.sigSNS signals with respect to the power of sim_sig_SrcActiv.sigSNS, to obtained desired signal-to-interference, signal-to-background-activity, and signal-to-measurement-noise ratios, respectively. These ratios are defined by the user using SETUP.SINR, SETUP.SBNR, and SETUP.SMNR variables, respectively, and are expressed in decibels [dB] using the following implementation: 
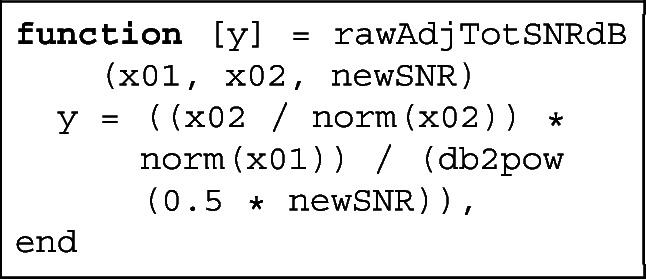
where db2pow is the Matlab function converting decibels to power.

#### Reconstruction Class

Class EEGReconstruction computes implemented spatial filters and applies them to the observed y_Pst simulated EEG signal. It also computes various fidelity measures of the reconstruced activity of sources of interest.

- EEGReconstruction:
  - setfilters — calculates matrices which are used in the process of reconstruction:
    - spatialfilterconstants — We compute some of constats used later in defining filters.
    - spatialfiltering — We compute all intermediate variables needed to calculate the filters.
    - spatialfilteringexecution — For every filter *W*(*θ*) given as a parameter to this function we are calculating *W*(*θ*)*y* using post-interval signal as *y*. Then we perform arfit for all reconstructed signals and obtain autoregression matrix. This matrix is necessary to calculate PDC and DTF measures.
    - spatialfilteringerroreva- luation — Function which calculates difference between original signal and reconstructed. For that it uses various measures: Euclidean metric and correlation coefficients to compare activity signals, MVAR coefficient matrices and PDC and DTF coefficient matrices.
    - vectorizerrorevaluation — Function that combines results in single array. Such uniform output of different error measures is later used in plotting.
  - printaverageresults — print table of comparison of different reconstruction filters.
  - save — save reconstructed filters.

### Plotting Class

The toolbox makes it possible to visualize results of experiments. User can plot results of simulation using EEGPlotting class which is specially prepared for this. 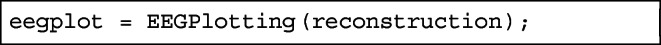


**Fig. 6:**
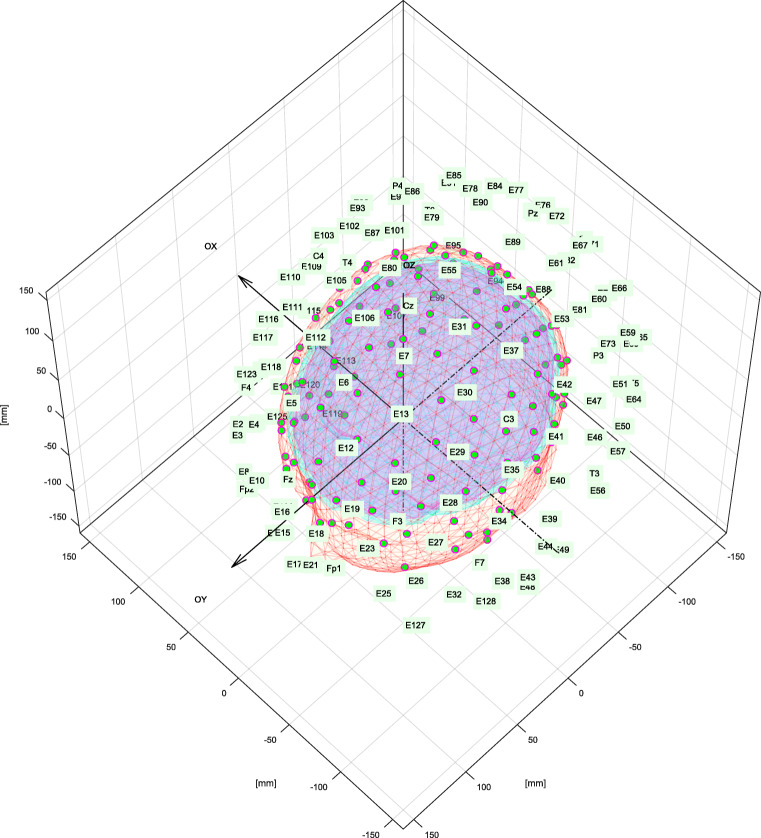
Volume conduction model essential components. Triangulation meshes representing brain, skull and scalp boundaries with electrode positions plotted on top of the scalp surface. This figure was generated using an instance of EEGPlotting class employing plotbrainoutermesh(), plotskulloutermesh(), plotscalpoutermesh(), plotelectrodepositioning() and plotelectrodelabels() methods

**Fig. 7:**
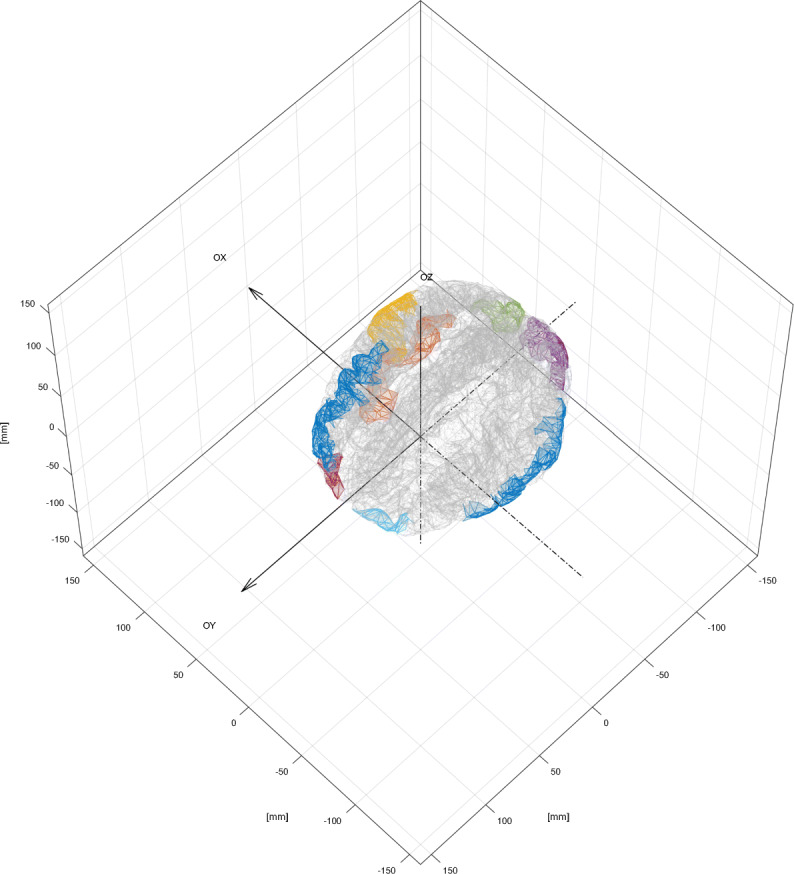
Cortex and ROIs. Detailed cortical surface triangulation with selected cortical patches. This figure was generated using an instance of EEGPlotting class employing textttplotcortexmesh() and textttplotROIvisualization() methods

**Fig. 8:**
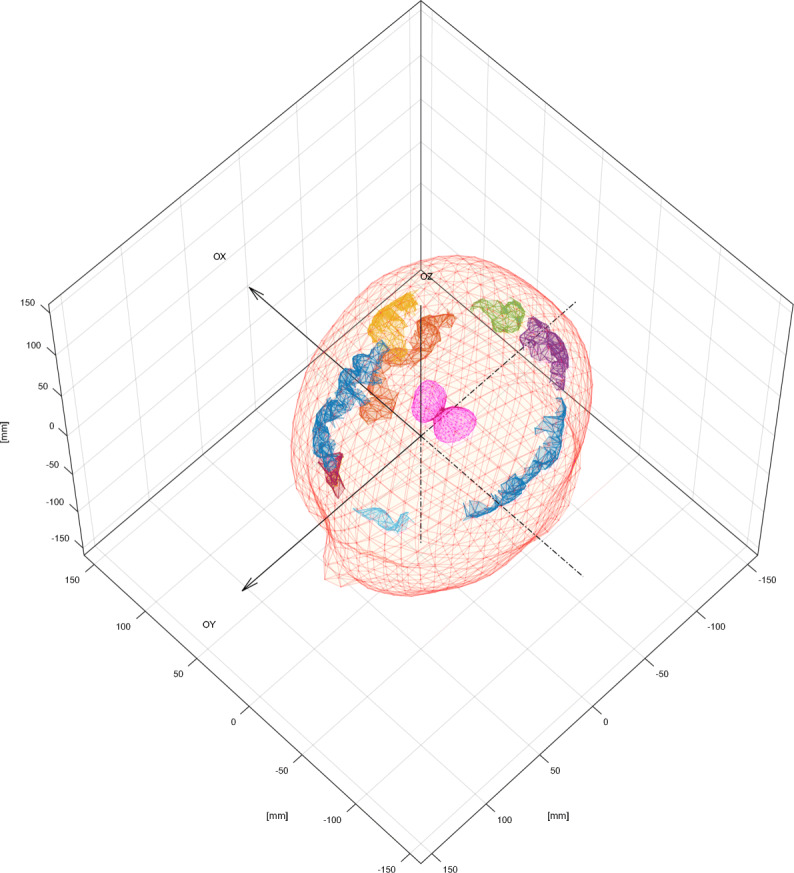
ROIs and thalami. Cortical patches selected as a candidate ROIs for source position with thalami mesh and scalp outer mesh. This figure was generated using an instance of EEGPlotting class employing plotROIvisualization(), plotdeepsourcesasthalami() and plotscalpoutermesh() methods

**Fig. 9:**
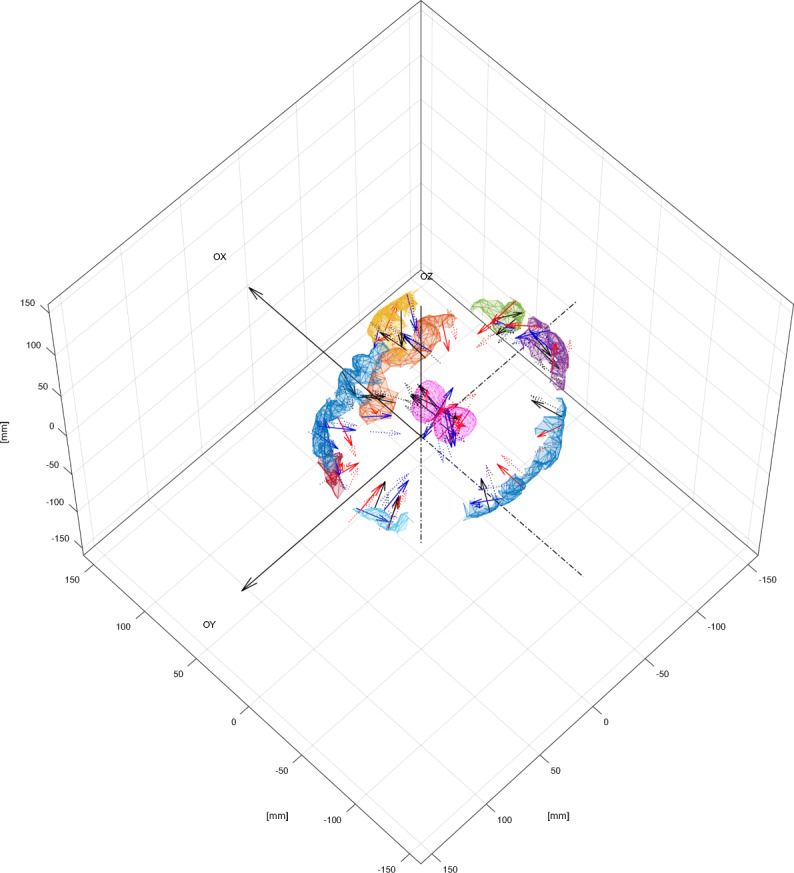
Bioelectrical activity positions and orientations. Cortical patches selected as a candidate ROIs for source position with thalami mesh. Vectors representing direction of the dipole moments for the sources of bio-electrical activity. Red lines represent the activity of interest; blue – the interfering activity and black – background activity. Solid lines represent original sources and dotted lines represent perturbed sources. Arrows representing dipole position and orientation are drawn not to scale. This figure was generated using an instance of EEGPlotting class employing plotROIvisualization(), plotdeepsourcesasthalami() and plotsourcevisualization() methods

Interesingly, there are many ways to facilitates visualization of the analysis and results. Jupyter functionality gives us a possibility to plot figures inside notebook (using %plot magic option): 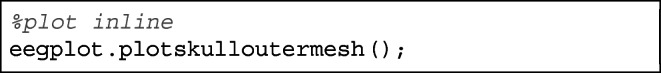


or to open it in Matlab interactive environment: 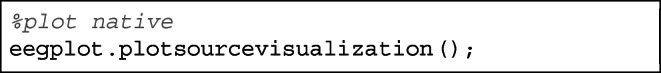


Toolbox supFunSim contains variety of plotting functions for different visualizations of results. Some of them are presented in Figs. 6, 7, 8 and 9.

Plot consists of layers that are generated by functions with self-explonatory names. E.g. function plotROIvisualization plots cortex, regions of interest. Function plotsourcevisualization plots mesh for ROIs on cortex, mesh for deep sources ROI, sources and cortex.

- EEGPlotting:
  - plotMVARmodelcoefficientmatrix mask — this method plots mask for MVAR model coefficient matrix.
  - plotPDCgraph — this method is used for plotting PDC profiles across sources of interest and interfering sources.
  - plotDTFgraph — this method is used for plotting DTF profiles across sources of interest and interfering sources.
  - plotMVARmodelcoefficientmatrix graph — this method plots composite MVAR model matrix for sources of interest, interfering and background sources.
  - ploterrortable — this method plots results of reconstruction as heatmap table.
  - plotdeepsourcesasicosahedron642 — this method for plotting of deep sources.
  - plotdeepsourcesasthalami — this method can be used for plotting of both thalami.
  - plotcortexmesh — this method plots cortex mesh.
  - plotbrainoutermesh — this method plots brain outer mesh.
  - plotskulloutermesh — this method plots skull outer mesh.
  - plotscalpoutermesh — this method plots sclap outer mesh.
  - plotelectrodepositioning — this method plots electrode positions.
  - plotelectrodelabels — this method plots electrode labels.
  - plotROIvisualization — this method plots ROIs based on generated meshes.
  - plotsourcevisualization — this method is used for source vizualization.

### Unit Test Class

Since this implementation is based on the previous one, which was done in Org-mode, authors have created a class EEGTest for unit tests.

In order to generate and distribute files into directories (necessary for the test) there is a make target test.

For example, unit tests can look like that: 
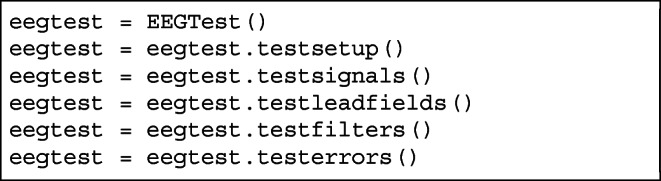


This class can be useful for developers who wants to extend our framework. This way they always can check whether it is still passes the compliance test.

## Sample Usage

### Generating Set of Parameters for Simulations

Generation of a new simulation requires preparation of a set of parameters. This is done by the EEGParameters class, in which the generate method is included. In the default version the dummy function is called, which returns the default parameter structure, but the user can always overwrite the function to create own version or change the configuration of the simulation and perform own experiments. Syntax for generating parameters is as follows: 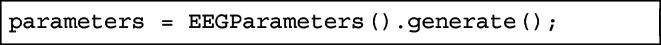


Several sample settings have been prepared. Functions setinitialvalues and setsnrvalues set up initial parameters and signal to noise ratios. Function smartparameters overwrites initial values and contains parameters for the sample run. Function testparameters contains parameters for unit test done by EEGTest. For that line containing this function must be uncommented.

Generated structure contains about 50 fields. All fields are listed in Table 1.

### Example Run Of Simulations

The input configuration structure from EEGParameters class contains options and parameters that specify how the stimulation will run. Once the user is satisfied with the parameter settings a sequence of simulations is ready to run, which may look, e.g., like that: 
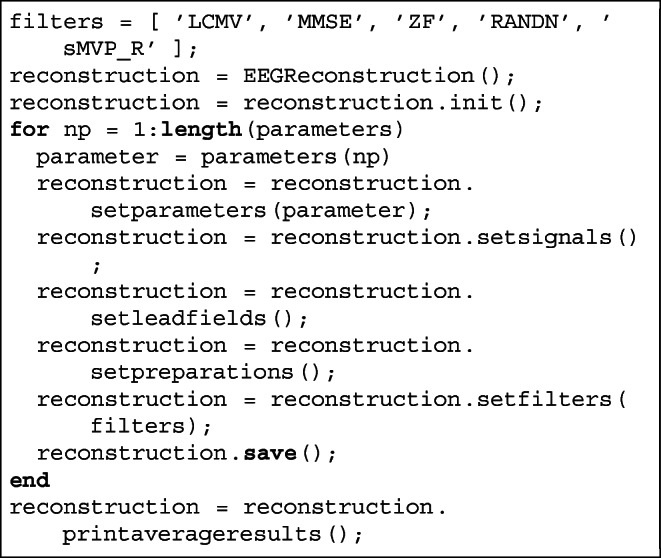


Here parameters were generated as described above. Selection of filters to compute the source reconstruction is done above in a very direct way. Filters are self-contained, i.e. they can run independently. What is worth noting, there are fifteen spatial filters available in the current version of supFunSim (including, e.g., classical LCMV), and more will be added.

All intermediate values of model variables along with the initial settings are stored in attributes SETUP and MODEL. Attribute RESULTS contains the scores of all the most important measures of errors for individual filters. Moreover, all meshes are kept in attribute MATS. The main reason is that meshes are loaded only once during all iterations. All that, as a part of the main object, can be saved in mat file and later restored. 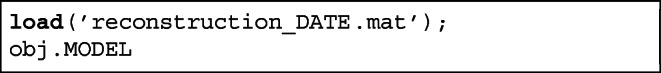


Here DATE is identifier of reconstruction, given by date of execution.

## Future Work

Some applications require combination of spatial filtering modeling of source activity in frequency domain. It is certainly worthwhile to add additional transformation such as Laplace transformation (Kayser and Tenke 2015) to avoid dependence of the reference. The choice of commercial Matlab toolbox may also be a limitation for some users and it will be worthwhile to convert the whole package to languages such as Python. Adaptive filters have not yet been implemented, therefore creation of on-line applications is not yet feasible.

## Information Sharing Statement

The source code of the toolbox is publicly available at https://github.com/nikadon/supFunSim as an Org-mode file, Jupyter notebook, and also as a plain Matlab source code.

## Appendix

### Implemented Spatial Filters

We denote the concatenated composite lead-field matrices of *H* and $H_{\mathfrak {i}}$ as $H_{\mathfrak {c}} := [H\ H_{\mathfrak {i}}]$, and similarly $q_{\mathfrak {c}} := [q^{t} q_{\mathfrak {i}}^{t}]^{t}.$ The covariance matrices of *q*, $q_{\mathfrak {c}}$, $q_{\mathfrak {b}} + n_{\mathfrak {m}}$, *y* are denoted by *Q*, $Q_{\mathfrak {c}}$, *N*, *Y*, respectively.

We selected for comparison the following spatial filters:

1. The LCMV filter, expressed as both (Moiseev et al. 2011)
  8 $$ W_{LCMV(R)} = (H^{t} R^{-1} H)^{-1} H^{t} R^{-1}, $$
  and Moiseev et al. (2015)
  9 $$ W_{LCMV(N)} = (H^{t} N^{-1} H)^{-1} H^{t} N^{-1}. $$
2. The nulling filter (Hui et al. 2010):
  10 $$ W_{NL} = [\mathbb{I}_{l}\ 0_{l\times k}] ({H_{c}^{t}} R^{-1} H_{c})^{-1} {H_{c}^{t}} R^{-1}. $$
3. The Wiener filter, defined as Kailath et al. (2000)
  11 $$ W_{F-MMSE} = QH^{t}R^{-1}, $$
  for the interference-free model, and Kailath et al. (2000)
  12 $$ W_{I-MMSE} = \mathbb{E}[q{q_{c}^{t}}] {H_{c}^{t}} R^{-1}, $$
  for the model in presence of interference.
4. The zero-forcing filter, defined as Kailath et al. (2000)
  13 $$ W_{ZF} = H^{\dagger}, $$
  where *H*^‡^ denotes pseudo-inverse of *H*.
5. The eigenspace-LCMV filters (Sekihara and Nagarajan 2008) exploiting projection of the signal covariance matrix *R* onto its principal subspace of the forms
  14 $$ W_{EIG-LCMV(R)} = W_{LCMV(R)} P_{R_{sig}}, $$
  and
  15 $$ W_{EIG-LCMV(N)} = W_{LCMV(N)} P_{R_{sig}}, $$
  where $P_{R_{sig}}$ is the orthogonal projection matrix onto subspace spanned by eigenvectors corresponding to $\lambda _{1} \geq {\dots } \geq \lambda _{sig}$ — the *sig* largest eigenvalues of *R*, where *sig* is the dimension of signal subspace.
6. The MV-PURE filters, defined as Piotrowski et al. (2019)
  16 $$ W_{F-MV-PURE}^{r} = P_{L_{r}^{(i)}} W_{LCMV(R/N)}, $$
  for the interference-free model, and Piotrowski et al. (2019)
  17 $$ W_{I-MV-PURE}^{r} = P_{K_{r}^{(i)}} W_{NL}, $$
  for the model in presence of interference. In the above expressions, $P_{L_{r}^{(i)}}$, for *i* = 1, 2, 3, are the orthogonal projection matrices onto subspaces spanned by eigenvectors corresponding to the *r* smallest eigenvalues of symmetric matrices $L^{(1)} = W_{LCMV(R)} R W_{LCMV(R)}^{t} - 2Q$, $L^{(2)} = W_{LCMV(R)} R W_{LCMV(R)}^{t}$, $L^{(3)} = W_{LCMV(N)} N W_{LCMV(N)}^{t}$, respectively; similarly, $P_{K_{r}^{(i)}}$, for *i* = 1, 2, 3, are the orthogonal projection matrices onto subspaces spanned by eigenvectors corresponding to the *r* smallest eigenvalues of symmetric matrices $K^{(1)} = W_{LCMV(R)}RW_{LCMV(R)}^{t} - 2Q$, $K^{(2)} = W_{LCMV(R)} R W_{LCMV(R)}^{t}$, $K^{(3)} = W_{LCMV(N)} N W_{LCMV(N)}^{t}$, respectively. Here, *Q* is the covariance matrix of sources of interest *q*.

The file EEGReconstruction.ipynb is richly commented using mathematical formulas, thanks to which it will be easy to find a concrete place of implementation of a specific filter.

### Configuration

Table 1 contains all configuration parameters. Some of the more complex parameters have been explained below the table.

Within SETUP, perhaps the most important are the parameters controlling configuration of activity of sources (SRCS), configuration of deep sources (DEEP), number of samples and realizations of the signal (n00, K00), presence of ERPs (ERPs), number of iterations (ITER), configuration of lead-fields perturbation (CUBE, CONE), signal to noise ratios, (SINR, SBNR, SMNR), and presence of signal components (SigPre, IntPre, BcgPre, MesPre, SigPst, IntPst, BcgPst, MesPst).

There are 7 structures containing head conduction model. They are listed in Table 2.

**Table 2:** MATS structure containing all meshes

Parameter	Description
sel_msh	structure with head compartments geometry (cortex)
sel_geo_deep_thalami	structure with mesh containing candidates for location of deep sources (based on *thalami*)
sel_geo_deep_icosahedron642	structure with mesh containing candidates for lacation of deep sources (based on *icosahedron642*)
sel_atl	structure with cortex geometry with (anatomical) ROI parcellation
sel_vol	structure with volume conduction model (head-model)
sel_ele	structure with geometry of electrode positions
sel_src	structure with all cortex lead-fields

## Footnotes

1 *“The great topmost sheet of the mass, that where hardly a light had twinkled or moved, becomes now a sparkling field of rhythmic flashing points with trains of traveling sparks hurrying hither and thither. The brain is waking and with it the mind is returning. It is as if the Milky Way entered upon some cosmic dance. Swiftly the head mass becomes an enchanted loom where millions of flashing shuttles weave a dissolving pattern, always a meaningful pattern though never an abiding one; a shifting harmony of subpatterns”.* – Charles S. Sherrington, Man on his nature. 1942

2 It can be download from http://neuroimage.usc.edu/bst/download.php?file=tempColin27_2012.zip and contains: tess_innerskull.mat, tess_outerskull.mat and tess_head.mat.
